# Dynamic monitoring serum tumor markers to predict molecular features of EGFR‐mutated lung cancer during targeted therapy

**DOI:** 10.1002/cam4.4676

**Published:** 2022-05-11

**Authors:** Zhuxing Chen, Liping Liu, Feng Zhu, Xiuyu Cai, Yi Zhao, Peng Liang, Limin Ou, Ran Zhong, Ziwen Yu, Caichen Li, Jianfu Li, Shan Xiong, Yi Feng, Bo Cheng, Hengrui Liang, Zhanhong Xie, Wenhua Liang, Jianxing He

**Affiliations:** ^1^ Department of Thoracic Surgery/Oncology, China State Key Laboratory of Respiratory Disease & National Clinical Research Center for Respiratory Disease the First Affiliated Hospital of Guangzhou Medical University Guangzhou China; ^2^ Department of General Internal Medicine, Sun Yat‐sen University Cancer Centre, State Key Laboratory of Oncology in South China Collaborative Innovation Centre for Cancer Medicine Guangzhou China; ^3^ Department of Respiratory Disease, China State Key Laboratory of Respiratory Disease & National Clinical Research Center for Respiratory Disease the First Affiliated Hospital of Guangzhou Medical University Guangzhou China

**Keywords:** circulating tumor DNA, epidermal growth factor receptor Thr790Met, lung cancer, oncogenic drivers, serum tumor markers

## Abstract

To reveal the correlation of dynamic serum tumor markers (STMs) and molecular features of epidermal growth factor receptor‐mutated (EGFR‐mutated) lung cancer during targeted therapy, we retrospectively reviewed 303 lung cancer patients who underwent dynamic STM tests [neuron‐specific enolase (NSE), carcinoembryonic antigen (CEA), carbohydrate antigen 125 (CA125), carbohydrate antigen 153 (CA153), the soluble fragment of cytokeratin 19 (CYFRA21‐1), and squamous cell carcinoma antigen (SCC)] and circulating tumor DNA (ctDNA) testing with a panel covering 168 genes. At baseline, patients with EGFR mutation trended to have abnormal CEA, abnormal CA153, and normal SCC levels. Additionally, patients with Thr790Met (T790M) mutation were more likely to have abnormal CEA levels than patients without T790M mutation. Among patients with secondary resistance to EGFR tyrosine kinase inhibitors (TKI), the dynamic STMs showed a descending trend in the responsive stage and a rising trend in the resistant stage. However, the changing slopes differed between T790M subgroup and the non‐T790M subgroup in individual STMs. Our study demonstrated that the combination of baseline levels and variations of STMs (including the responsive stage and resistant stage) can be suggestive of secondary EGFR‐T790M mutation [area under the curve (AUC) = 0.897] and that changing trends of STMs (within 8 weeks after initiating the TKI therapy) can be potential predictors for the clearance of EGFR ctDNA [AUC = 0.871]. In conclusion, dynamic monitoring STMs can help to predict the molecular features of EGFR‐mutated lung cancer during targeted therapy.

## Graphical abstract

Predicting the molecular features of cancer at an earlier time would be conducive to precise clinical diagnoses and treatments. To our knowledge, there is still no research on whether dynamic monitoring tumor markers could predict the molecular features of lung cancer during targeted therapy. In this study, we demonstrated that dynamic lung‐cancer‐relate STMs can be effective predictors for secondary EGFR T790M mutation and the clearance of EGFR ctDNA during targeted treatment.

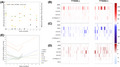

## 1 INTRODUCTION

Lung cancer has become the most commonly diagnosed cancer and the leading cause of cancer death in the world.^1^ Various oncogenic drivers have been discovered since 1977, including EGFR.^2^, ^3^, ^4^, ^5^ Meanwhile, the use of targeted drugs has significantly improved the prognosis of patients with targetable mutations.^2^ However, different treatment strategies may result in different outcomes, and secondary resistance might be induced during the treatment.^6^

To timely adjust the therapeutic strategy of lung cancer, the detections of biomarkers (e.g., tissue biopsy, liquid biopsy, serum tumor markers) are employed to monitor the dynamic tumor activity.^7^, ^8^, ^9^ Although the golden standard for molecular profiling is tissue biopsy, patients limited by their poor physical conditions or inoperable lesion locations cannot undergo surgery or fine‐needle aspiration biopsy.^10^ Also, the insufficient tissue specimens, potential risks of surgery and tumor transmission, and dynamic sampling add difficulty in using tissue biopsy as a tool for dynamic monitoring.^10^, ^11^, ^12^, ^13^ For cases with multiple primary lesions, a single lesion biopsy is insufficient to reveal tumor activity comprehensively due to tumor heterogeneity.^11^, ^12^ Thus, liquid biopsy has been developed and has become an important complementary tool for biopsy.^9^ However, liquid biopsy faces challenges in widespread clinical application due to its technical limitations and high costs. A more cost‐effective alternative tool to monitor molecular features of lung cancer during targeted therapy is needed.

According to previous studies, STM testing helps diagnose suspected cancer and unknown primary tumor and evaluate anti‐tumor therapy.^14^, ^15^, ^16^, ^17^ The most commonly used STM testing for lung cancer includes neuron‐specific enolase (NSE), carcinoembryonic antigen (CEA), cancer antigen 125(CA125), carbohydrate antigen 153(CA153), the soluble fragment of cytokeratin 19 (CYFRA21‐1), and squamous cell carcinoma antigen (SCC).^7^, ^8^, ^14^, ^17^ Furthermore, combining CA125 with SCC could predict EGFR mutations.^18^ However, to our knowledge, no study was conducted to predict the molecular features during the targeted treatment by STMs. Therefore, we aim to predict molecular features of EGFR‐mutated lung cancer, including the emergence of secondary EGFR‐T790M mutation and the clearance of EGFR ctDNA, by dynamically monitoring STMs during targeted therapy.

## 2 MATERIALS AND METHODS

### 2.1 Study design and patient cohort

We retrospectively reviewed 303 Chinese patients with lung cancer who received first‐line targeted therapy with a 168 genes panel sequencing developed by Burning Rock Dox between September 2015 and July 2019 at the First Affiliated Hospital of Guangzhou Medical University. Their longitudinal plasma or tissue specimens were collected at baseline (within 15 days before starting first‐line targeted therapy) and throughout the treatment. Besides, patients must have had six STMs tested at baseline every month, including NSE, CEA, CA 125, CA 153, CYFRA 21‐1, SCC. ctDNA and STMs sampling were collected during the same visit. CT scan was also done in all patients every 1–4 months (depending on patients' symptoms, the timing after targeted therapy, and patient's economic capability) to evaluate treatment efficacy according to RECIST 1.1. The targeted therapy was the first‐ or second‐generation EGFR‐TKI as first‐line therapy in all patients.

**FIGURE 1:**
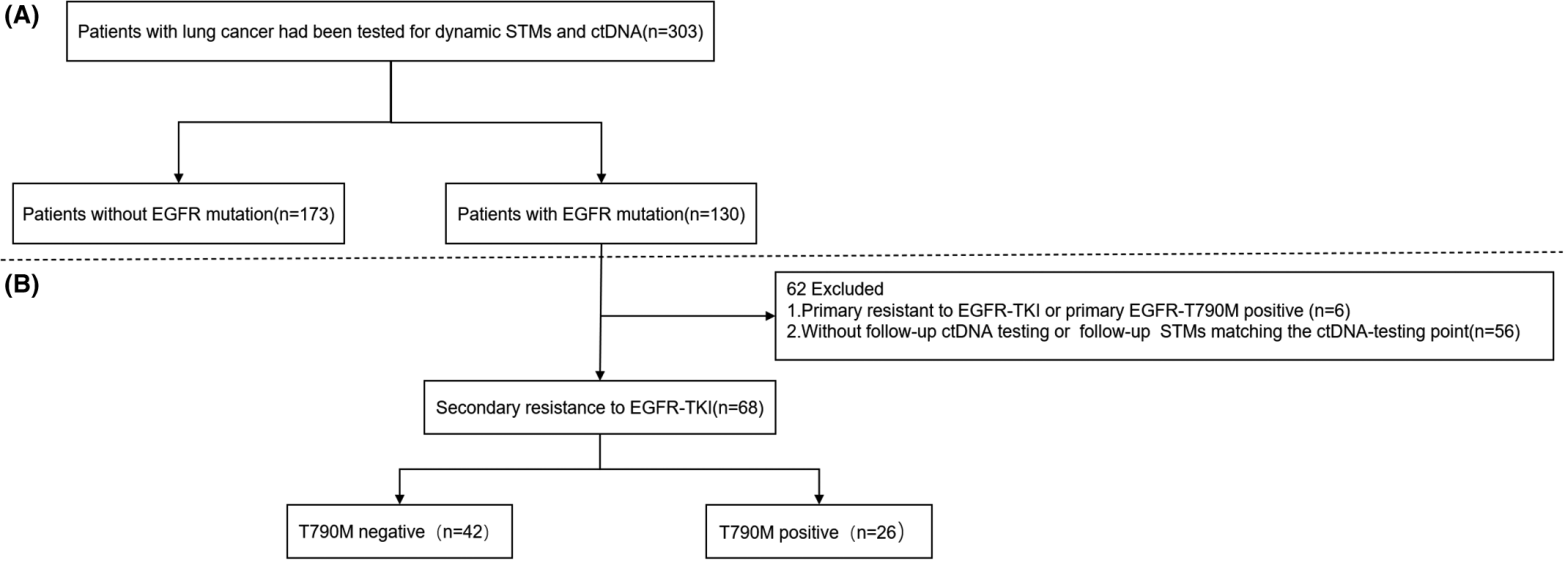
Study design and algorithm of patient selection

To evaluate the predicting ability of STMs on the emergence of secondary resistance and clearance of EGFR ctDNA, we further excluded 62 patients from the EGFR group (*n* = 130) for the following reasons: (1) 6 patients were excluded because they showed primary resistance to EGFR TKI or primary EGFR T790M positive; (2) 56 patients were excluded because they had not conducted follow‐up ctDNA testing or did not have STMs done at the same time as the corresponding ctDNA (Figure 1).

### 2.2 Next‐generation sequencing (NGS) library preparation and capture‐based targeted DNA sequencing

A total of 10 mL of peripheral blood was used as input material for library preparation. The supernatant following centrifugation for 10 min at 2000*g* at 4 °C was transferred to a new tube and centrifuged again at 16,000*g* at 4°C for 10 min. Subsequently, circulating free DNA was isolated from plasma using the QIAamp Circulating Nucleic Acid kit (Qiagen). Quality was verified by using the Qubit 2.0 Fluorimeter with the dsDNA HS assay kits (Life Technologies). A minimum of 50 ng of cfDNA is required for NGS library construction. Circulating free DNA was extracted using the QIAamp Circulating Nucleic Acid kit (Qiagen). Then subjected to end repair, phosphorylation and adaptor ligation. Fragments of size 200–400 bp were selected by AMPure beads (Agencourt AMPure XP Kit). Targeted DNA was captured, selected, and amplified. Quality of the fragments was assessed by using a bioanalyzer high‐sensitivity DNA assay. Indexed samples were sequenced in one lane on a Nextseq500 sequencer (Illumina, Inc.) with pair‐end reads. The mean coverage depth was 11,828×. Our assay captures 168 genes that are listed in the Gene list. The sequencing coverage and quality statistics, as well as the exact EGFR‐T790M mutation status are for each sample listed in the Table S1.

### 2.3 Sequence data analysis

Sequence reads were mapped to the human genome (hg19) using BWA‐MEM (v.0.7.10) with default parameters. Following GATK v.3.2, PCR duplicates were first removed and subsequently realigned and recalibrated. Variant calling was performed using MuTect and VarScan. We filtered variants by using the VarScan filter pipeline, with loci with depth less than 100 filtered out. We further filtered out mutations tending to be false positives, retaining only INDELs supported by at least two supporting reads and SNVs supported by at least eight reads. In addition, according to the ExAC, 1000 Genomes, ESP6500SI‐V2 and dbSNP database, we excluded SNPs with population frequency over 0.1% from further analysis. The high‐quality variants were annotated with SnpEff v3.6. DNA translocations were identified using both Tophat2 and Factera 1.4.3. White blood cells were used as a reference to filter out germline mutations.

### 2.4 STMs measurement

STMs were detected using a commercial chemiluminescence immunoassay kit (Abbott Laboratories). Blood samples from all patients were obtained through peripheral venipuncture before the first TKI treatment for baseline evaluation, and every month during targeted therapy for monitoring. The following thresholds were considered the upper limits of normal: NSE, 16.3 mg/L; CEA, 5 mg/L; CA 125, 35 U/mL; CA 15–3, 25 U/mL; CYFRA 21‐1, 3.3 ng/mL; SCC, 1.5 ng/mL. Accordingly, tumor marker values above these thresholds were considered abnormal

### 2.5 Statistical analysis

We divided the responsive and resistant stages according to the changes in tumor size and STMs. Responsive stage: CT scan showed that the tumor size continued to decrease, and at least half of the STMs continued to decline; Resistant stage: at least two STMs change from continuous decline to continuous increase. In order to calculate the slope of the responsive stage and the resistant stage, three critical timepoints were set up, respectively. Timepoint 1: the last STMs detection before the first‐line treatment began; timepoint 2: the lowest value of STMs in the responsive stage, which was also the endpoint of the responsive stage or the start point of the drug resistance stage; timepoint 3: the peak value of STMs in the drug resistance stage before second‐line treatment. The calculation formula for each patient was as follows: The slope of the responsive stage = (STM value at timepoint 2 – STM value at timepoint 1)/(timepoint 2 – timepoint 1); The slope of the resistant stage = (STM value at timepoint 3 – STM value at timepoint 2)/(timepoint 3 – timepoint 2).

All statistical tests were bilateral with a significance level of 0.05 conducted in IBM SPSS software (version 25.0). The differences in the distribution of categorical variables across groups were assessed using the Fisher exact test or chi‐squared test. Heat maps were drawn in GraphPad Prism 8.0 as a visualization method to assess the variation of STMs at baseline, responsive stage, and resistant stage in the subgroup of EGFR T790M mutation and non‐EGFR T790M mutation. The accuracy of the multivariate predictors of molecular mechanisms was measured using the area under the receiver operating characteristic (ROC) curve.

## 3 RESULTS

### 3.1 General baseline characteristics of patients

Within the 303 eligible patients in our study, general baseline characteristics, including gender, age, histological type, clinical staging, oncogene type, and percentages of patients with an elevated STM regarding each STM, are summarized in the Table 1 and the Table S2.

**TABLE 1:** General baseline characteristics of patients

Characteristic	No. of patients (*n* = 303)	Percentage (%)
Gender		
Male	189	62.4%
Female	114	37.6%
Age		
Median (range)	63 (23–88)	
Histological type		
Adenocarcinoma	274	90.4%
Squamous carcinoma	13	4.3%
Adenosquamous carcinoma	1	0.3%
SCLC	4	1.3%
Others^a^	11	3.6%
Clinical stage		
III	63	20.8%
IV	240	79.2%
Oncogenic types		
EGFR	130	42.9%
TP53	159	52.5%
ALK	30	9.9%
MET	21	6.9%
KRAS	36	11.9%
ROS1	11	3.6%
BRAF	12	4.0%
ERBB2	16	5.3%
RET	11	3.6%
NSE		
Elevated	209	69.0%
Normal	94	31.0%
CEA		
Elevated	197	65.0%
Normal	106	35.0%
CA125		
Elevated	172	56.8%
Normal	131	43.2%
CA153		
Elevated	140	46.2%
Normal	163	53.8%
CYFRA21‐1		
Elevated	236	77.9%
Normal	67	22.1%
SCC		
Elevated	102	33.7%
Normal	201	66.3%

^a^ Others include epitheliomatoid carcinoma, complex small cell carcinoma, and other unknown pathological types of lung cancer.

### 3.2 Routine STMs are associated with oncogenic types

After grouping the patients according to the oncogenic types, the differences in the above variants between patients harboring mutated type and wild type oncogenes were investigated and summarized in the Table S2. Patients with TP53 mutations and EGFR mutations accounted for the largest [159(52.5%)] and the second largest [130(42.9%)] proportion. EGFR mutations were significantly associated with female gender (57.9% vs. 33.9%; *p* <0.001), abnormal CEA level (48.7% vs. 32.1%; *p* <0.05), abnormal CA153 level (52.1% vs. 35.0%; *p* <0.05), and normal SCC level (51.2% vs. 26.5%; *p* <0.001). In comparison, TP53 mutations were significantly associated with male gender (57.1% vs. 44.7%; *p* <0.05), abnormal NSE level (56.9% vs. 42.6%; *p* <0.05), normal CYFRA21‐1 level (55.9% vs. 40.3%; *p* <0.05). Besides, other oncogenic types including ALK, MET, KRAS, ROS1, BRAF, ERBB2, and RET were analyzed (Table S2). Among all EGFR‐mutated patients (*n* = 130), patients with T790M mutation (*n* = 33) were more likely to have an abnormal CEA level (31.3% vs. 8.8%; *p* <0.05) (Table 2).

**TABLE 2:** Characteristics comparisons between EGFR muted patients with and without T790M

Characteristic	EGFR mutated patients (*n* = 130)		
	T790M‐negative (*n* = 97)	T790M‐positive (*n* = 33)	*p*‐value
Gender			0.761
Male	47 (73.4)	17 (26.6)	
Female	50 (75.8)	16 (24.2)	
Age			
Median	64	56	
Range	33–87	34–83	
NSE			0.642
Elevated	66 (75.9)	21 (24.1)	
Normal	31 (72.1)	12 (27.9)	
CEA			**<0.01**
Elevated	66 (68.8)	30 (31.3)	
Normal	31 (91.2)	3 (8.8)	
CA125			0.551
Elevated	56 (72.7)	21 (27.3)	
Normal	41 (77.4)	12 (22.6)	
CA153			0.316
Elevated	52 (71.2)	21 (28.8)	
Normal	45 (78.9)	12 (21.1)	
CYFRA21‐1			0.508
Elevated	76 (76)	24 (24)	
Normal	21 (70)	9 (30)	
SCC			0.056
Elevated	24 (88.9)	3 (11.1)	
Normal	73 (70.9)	30 (29.1)	
Pathology			
Adenocarcinoma	92 (70.8)	32 (24.6)	
Squamous carcinoma	2 (1.5)	0 (0)	
Adenosquamous carcinoma	0 (0)	0 (0)	
SCLC	1(0.8)	0 (0)	
Others^a^	2 (1.5)	1 (0.8)	
Gross	97 (74.6)	33 (25.4)	

Values presented are *n* (%) unless otherwise noted.

^a^ Others include epitheliomatoid carcinoma, complex small cell carcinoma, and other unknown pathological types of lung cancer.

### 3.3 Dynamic changing patterns of STMs during EGFR‐TKI therapy

**FIGURE 2:**
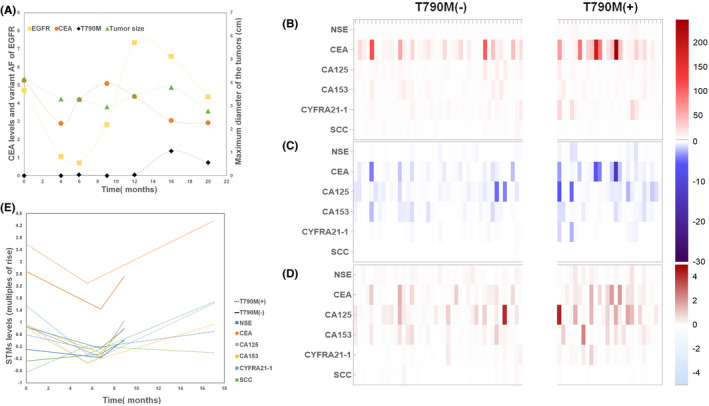
Dynamic changes of STMs, ctDNA, and tumor size. Changing trends of CEA (the logarithm of the CEA value), EGFR, and tumor size during EGFR‐TKI treatment. Each line represents the average value of patients in the corresponding group. Since the follow‐up times for each test were not synchronous, we used intervals of 2–4 months to compare the dynamic changes between groups. The actual follow‐up times for each test are controlled within plus or minus 1 month of the corresponding timepoint. We divided the whole treatment process into seven timepoints: 0, 4, 6, 9, 12, 16, and 20 months after the initiation of targeted therapy. (A). Heat maps and line charts of the STMs in the T790M subgroup and the non‐T790M subgroup; Multiples of the rise of the STMs at baseline(B). Decrease slope of the STMs in the responsive stage(C). Increase slope of the STMs in the resistant stage(D). The overall trend (The logarithm of the STM level) of STMS in the non‐T790M resistant subgroup (dotted lines) and the T790M resistant subgroup (solid lines) (E)

In order to explore the dynamic changes of STMs, ctDNA, and tumor size during the targeted therapy, we compared them at intervals of 2–4 months (Figure S1, Figure 2A). We found that the dynamic changing patterns of CEA value, variant allele frequency (AF) of EGFR, and tumor size were similar, but the transition time was different. The STMs were the first to change from a continuous decline to a continuous rise. Compared with tumor size, the transition time of STMs and EGFR came earlier. Additionally, the transition time of STMs appeared at least 2 months earlier than that of EGFR, both from decline to increase and from increase to a decline. Furthermore, the secondary T790M mutation was detected 2 months later than the STMs turning point, indicating that STMs might be capable of predicting the occurrence of drug resistance earlier than ctDNA testing. (Figure 2A, Table S3).

To further explore the dynamic variations of STMs in the EGFR‐mutated population who developed secondary resistance during the targeted therapy, we excluded 62 patients with primary resistance to EGFR‐TKI, baseline T790M positive, or incomplete follow‐up testing (Figure 1B). The remaining 68 patients were considered to harbor secondary resistance to EGFR‐TKI because they had an initial response to EGFR‐TKI but developed resistance afterward. Besides, these 68 patients were negative for T790M at baseline, and they were further divided into the T790M subgroup (*n* = 26) and the non‐T790M subgroup (*n* = 42) regarding the secondary resistance molecular mechanism (Figure 1B). Generally, NSE, CEA, CA125, CA153, CYFRA21‐1 levels were higher in the T790M subgroup than the non‐T790M subgroup throughout the course and showed different downturns and upturns during the responsive and resistant stage, respectively (Figure S2). Furthermore, the dynamic changing patterns of STMs during the entire treatment courses, evaluated by the multiples of rising at baseline, the decline slope, and the rising slope of STMs, differed between the T790M and the non‐T790M subgroups, also between each STM even within the same group (Figure 2B–E, Figure S3).

At baseline, NSE, CEA, CA125, CA153, and CYFRA21‐1 in the population with T790M mutation were higher than those without T790M mutation, among which CEA and CA125 were the most prominent. Notably, the SCC level was higher in the non‐T790M subgroup than the T790M subgroup, distinguishing it from other STMs (Figure 2B, Figure S3A).

In the responsive stage, CEA, CA125, CA153, and CYFRA21‐1 had a greater decline slope in the population with T790M mutation than those without T790M mutation, among which CEA and CA125 were the most prominent. However, the NSE and SCC levels did not decrease in patients without T790M mutation and even experienced an increase in population with T790M mutation (Figure 2C, E, Figure S3B).

In the resistant stage, CEA, CA125, CA153, and CYFRA21‐1 had a greater rise slope in the population with T790M mutation than those without T790M mutation, among which CEA and CYFRA21‐1 were more evident than CA125 and CA153. Significantly, consistent with the baseline and responsive stage, SCC had a distinctive changing pattern in the resistant stage. It increased more in the population without T790M mutation and even decreased in those with T790M mutation (Figure 2D, E, Figure S3C).

To summarize, the dynamic changes of STMs differed between the two subgroups and between each STM, consistently demonstrated by the heat maps and the line charts in Figure 2.

### 3.4 STMs are potential predictors of the molecular features of EGFR‐mutated lung cancer

**FIGURE 3:**
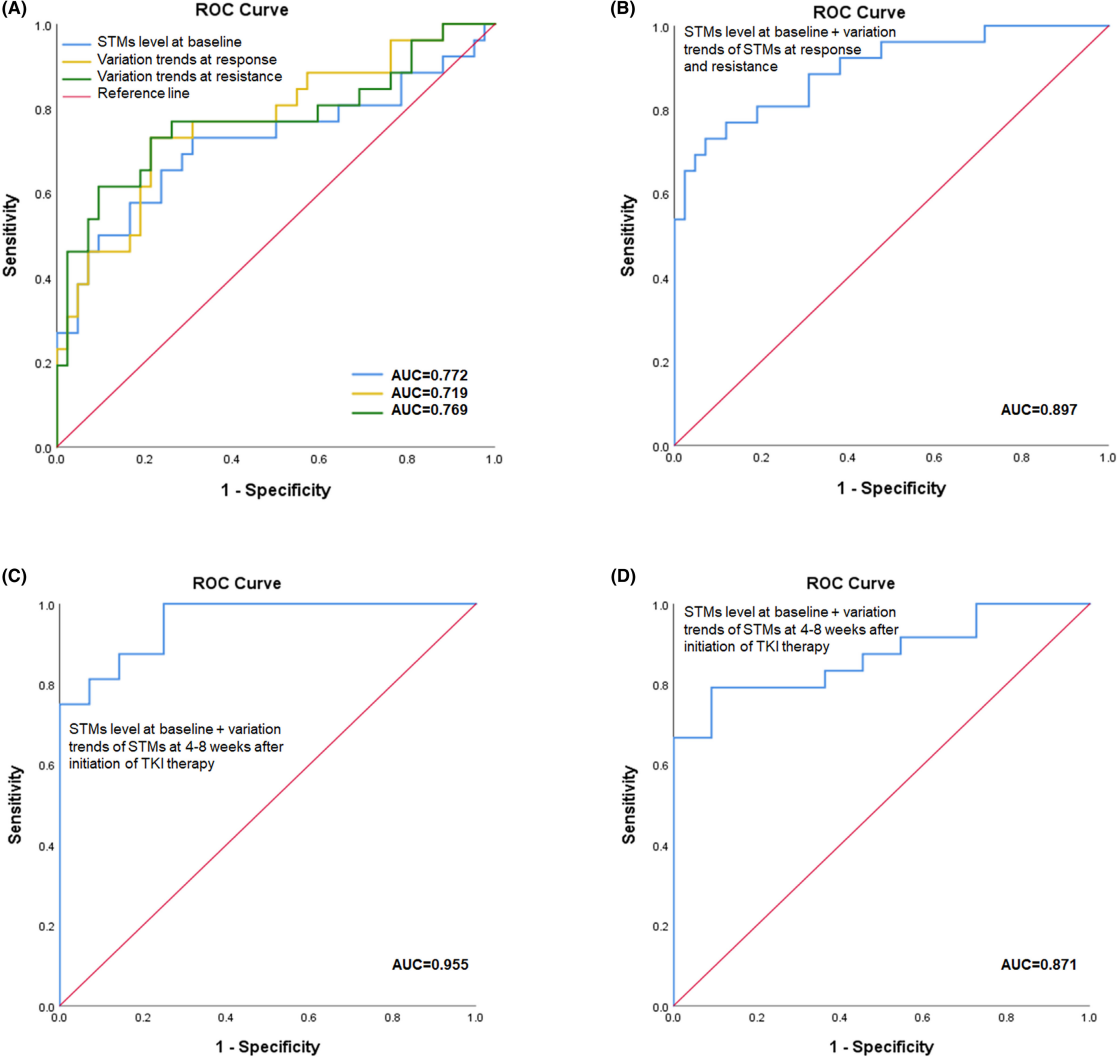
ROC curves of the STMs for predicting secondary EGFR‐T790M mutation and clearance of EGFR ctDNA. ROC curves of the STM level at baseline, decrease slope of the STMs in response and increase slope of the STMs in resistance respectively for predicting secondary EGFR‐T790M mutation(A). ROC curves of the combination of three factors for predicting secondary EGFR‐T790M mutation (B). ROC curves of the combination of the STM level at baseline and variation trends of the STMs at 4 to 8 weeks after initiation of targeted therapy for predicting secondary EGFR‐T790M mutation (C). ROC curves of the combination of the STM level at baseline and decrease slope of the STMs at 4 to 8 weeks after initiation of targeted therapy for predicting clearance of EGFR‐ctDNA (D)

We applied the ROC curves analysis to evaluate the value of dynamic STMs in predicting secondary EGFR‐T790M mutation. When the STM levels (The measured value divided by the upper limit of the respective standard range) at baseline were used, the AUC was 0.772[95% confidence interval (CI): 0.645,0.899]. In addition, AUC for the declining slopes in responsive stage was 0.719[95% CI: 0.581,0.857], and AUC for the rising slopes in the resistant stage was 0.769[95% CI: 0.650,0.889] (Figure 3A). When combining these three factors, the AUC was 0.897[95% CI: 0.818,0.975] (Figure 3B).

Notably, the ROC curve yielded an AUC of 0.955[95% CI: 0.901,1.000] when the above three factors were combined with a cutoff period from baseline to 4–8 weeks after initiation of targeted therapy (Figure 3C). When the same factors and period setup were used to predict the clearance of EGFR ctDNA, the ROC curve yielded an AUC of 0.871 [95% CI: 0.756–0.986] (Figure 3D). In comparison, using the combination of the baseline levels and the variation trends of STMs to predict other mutations, such as TP53, yielded unsatisfying AUC results (Figure S4).

## 4 DISCUSSION

Over the past decade, targeted therapy has become the mainstay of therapeutic regimens in lung cancer, especially EGFR‐mutated type. The targeted therapy application has extended from advanced lung cancer to early resectable lung cancer in staging, from adjuvant therapy to neoadjuvant therapy in therapeutic strategies.^19^, ^20^ Therefore, effectively monitoring the dynamic molecular features of lung cancer is an essential prerequisite for clinical decision‐making.

Somatic activating mutations in EGFR are the most common oncogenic driver in non‐small cell lung cancer (NSCLC), which have benefited from EGFR‐targeting therapies.^21^ Compared with radiation and chemotherapy, the response rate (60%–80%) of EGFR TKI for patients with EGFR mutant subtype (Exon19 deletion and Leu858Arg) is significantly higher than that of patients with EGFR wild type (10%–20%).^22^, ^23^ Despite the success of EGFR TKIs in EGFR mutant lung cancer, all patients eventually develop acquired resistance to these therapies.^6^ The prototypical mutation leading to EGFR‐TKI resistance in NSCLC is the EGFR‐T790M mutation, found in ≥50% of patients with acquired resistance to early‐generation EGFR TKIs.^24^, ^25^ Compared with patients without EGFR‐T790M mutation, the median progression‐free survival of patients with secondary T790M was significantly shorter. Some studies have shown that T790M can be detected 2–4 months before clinical progressive disease.^26^, ^27^, ^28^, ^29^ The standard strategy for T790M‐mediated resistance is to use the third generation of EGFR‐TKIs, such as Osimertinib. Therefore, it is helpful to develop methods to identify molecular progress before clinical progress, which may prompt more profound follow‐up and potential treatment adjustments.

Previous studies suggested that resistance mechanisms can usually be divided into pre‐adaption (pre‐existence) or post‐adaption (directed adaption as a response to directed choice). Pre‐adaption suggests that mutations might exist before the treatment but could not be revealed by current sequencing techniques.^30^, ^31^

STMs are tumor markers widely applied to screen unidentified tumors and monitor the activity of tumors, among which NSE, CEA, CA125, CA153, CYFRA21‐1, and SCC have been proved to be vital biomarkers related to lung cancer.^7^, ^8^, ^32^ The dynamic change of STMs from baseline has prognostic value for advanced NSCLC patients. High levels of the associated STMs were found to be significant as a predictive marker for early relapse,^33^ progression,^34^ effect of treatment,^35^ or worse survival.^36^, ^37^ In contrast, the decrease in associated STM levels was associated with favorable clinical outcomes.^7^, ^38^ In addition, compared with ctDNA, STMs can be conducted in a timely manner with lower costs in country‐level or provincial‐level hospitals. Therefore, this study aimed to explore a supplementary method based on STMs to reveal the molecular features of EGFR‐mutated lung cancer during targeted therapy.

Jin et al. reported that the EGFR‐mutation rate increased as serum CEA level increased,^39^ and Wang et al. reported that the EGFR mutations were associated with a normal serum SCC level.^18^ Besides, Bearz et al. reported that CA153 could serve as a reliable predictor of response to EGFR inhibitors in patients with bronchioloalveolar carcinoma.^40^ All the above evidence indicates that serum CEA, CA153, and SCC are correlated with EGFR mutation or the effect of EGFR TKI, which was confirmed in our study. We further explored the association between STMs and ctDNA follow‐ups and found that abnormal baseline CEA level was associated with secondary EGFR‐T790M mutations. However, the ROC yielded an AUC of 0.614, which means that individual static tumor markers cannot yield a satisfying predicting performance in this situation.

Different STMs often represent different pathological types of cancer, while pathological types of cancer are associated with different gene mutations.^41^, ^42^ Moreover, the conversion of dominant STMs sometimes might be suggestive of tumoral transformation, and the STM levels are often related to the tumor mutational burdens.^43^, ^44^ In addition to the acquired T790M mutation, the transformation from adenocarcinoma to other components is one of many mechanisms of acquired resistance to an EGFR TKI.^45^ Our results showed that compared with the T790M subgroup and the non‐T790M subgroup, SCC showed a distinctive pattern from other tumor markers at baseline, in the responsive phase, or in the resistant phase. Likewise, NSE also had a similar changing pattern in the responsive phase and the resistant phase. Previous studies have shown that increased SCC is associated with lung squamous cell carcinoma, and increased NSE is associated with small cell lung cancer.^41^, ^46^ Therefore, our results suggest that when lung cancer patients with EGFR mutation show increased NSE and SCC in the responsive stage of EGFR TKI, the mechanism of secondary drug resistance may be the gradual differentiation of lung adenocarcinoma into other components, rather than the acquired T790M mutation. However, we could not find relevant studies on the association between these two markers and EGFR‐TKI resistance, which is worth further exploration.

Furthermore, these may also suggest that applying static STMs as indexes is not informative enough to predict molecular features, and dynamic monitoring STMs might better reveal tumor molecular variations. In our study, the ROC yielded an AUC of 0.772 when all baseline STM levels were used to predict secondary EGFR‐T790M, which was significantly higher than the predictive accuracy of baseline CEA alone. Furthermore, the AUC reached 0.897 when we combined baseline STMs and all variations of STMs before the second‐line treatment to predict secondary EGFR‐T790M.

To enhance the practical value of our study, we also explored the predicting performance of STMs using a cutoff period from the baseline to 8 weeks after initiation of targeted therapy so that strategy adjustments can be taken in the early stage of the treatment. The ROC yielded an AUC of 0.955 when the combination of baseline STM level and changing trends of STMs within 8 weeks after targeted therapy was considered a predictor for secondary EGFR‐T790M. Significantly, the AUC was 0.871 when we explored the predictive accuracy of STMs within 8 weeks after targeted therapy for clearance of EGFR ctDNA after treatment. Therefore, dynamic STMs can indicate molecular features in EGFR‐mutated lung cancer, including the emergence of secondary EGFR‐T790M and the EGFR ctDNA clearance.

Our research provides values for guiding clinical work: First, STMs can be expected as a new strategy to predict the molecular features and the efficacy of targeted treatment at the early stage of the treatment, which can timely provide more precise guidance for clinical treatment schemes. Second, STM testing is non‐invasive and overcomes the drawbacks associated with tissue or liquid biopsies. It also helps clinicians estimate the appropriate timing for ctDNA sequencing to avoid excessive medical care burdens. To note, the purpose of our study is not to advocate that STMs can replace tissue or liquid biopsy. Instead, it complements the clinical judgment of clinicians, given a high false‐negative rate of ctDNA.^47^

There are several limitations to our study. First, as a single‐center retrospective study, the selection bias could not be avoided entirely, which may limit the accuracy and comprehensiveness of the results. Second, the sample size was relatively small to make a comprehensive prediction comparison of other driver genes and molecular mechanisms of resistance. Further research with larger sample size and multi‐center testification is warranted. Finally, though SuperARMS technology can overcome tumoral heterogeneity compared with the tissue biopsy, its higher false‐negative rate should be considered in clinical application.^47^ Future prospective trials are needed to investigate the predictive accuracy of STMs for other molecular mechanisms.

In conclusion, this is the first study based on STMs to predict the molecular features of EGFR‐mutated lung cancer during the targeted therapy, including clearance of targeted EGFR ctDNA and the emergence of secondary EGFR T790M. The changing trends of STMs varied in different oncogenic types and evolution stages of lung cancer. Therefore, we highlight that lung‐cancer‐related STMs (NSE, CEA, CA125, CA153, CYFRA21‐1, SCC) could be potential predictors of resistance and prognosis of lung cancer treated with targeted therapy. Further investigations are required to evaluate the intrinsic relationship between STMs and gene mutations.

## DECLARATION OF INTEREST

The authors declare no competing conflict of interest.

## AUTHOR CONTRIBUTIONS

ZX Chen, WH Liang, JX He, and ZH Xie designed the concept and experiments; ZX Chen, WH Liang, and LP Liu collected the data and did the analysis. ZX Chen, WH Liang, LP Liu, F Zhu, and XY Cai prepared the manuscript draft. ZX Chen, WH Liang, LP Liu, and ZH Xie revised the manuscript. All the authors reviewed and approved the final proof.

## ETHICS STATEMENT

This study was approved by the Ethics Committee of the First Affiliated Hospital of Guangzhou Medical University.

## Supporting information


FigureS1‐S4

Table S1

Table S2

Table S3

## Data Availability

The data that support the findings of this study are available from the corresponding author upon reasonable request.
