# Supplementary material for: Dynamic monitoring serum tumor markers to predict molecular features of EGFR‐mutated lung cancer during targeted therapy
Source: Cancer Med. 2022 May 11;11(16):3115–25. doi: 10.1002/cam4.4676 (PMC9385589; doi:10.1002/cam4.4676)
Supplement: Supplementary file 1 — FigureS1‐S4 [file CAM4-11-3115-s002.docx]

Figure S1. Changing trends of the tumor size, STMs, and ctDNA during EGFR-TKI treatment.


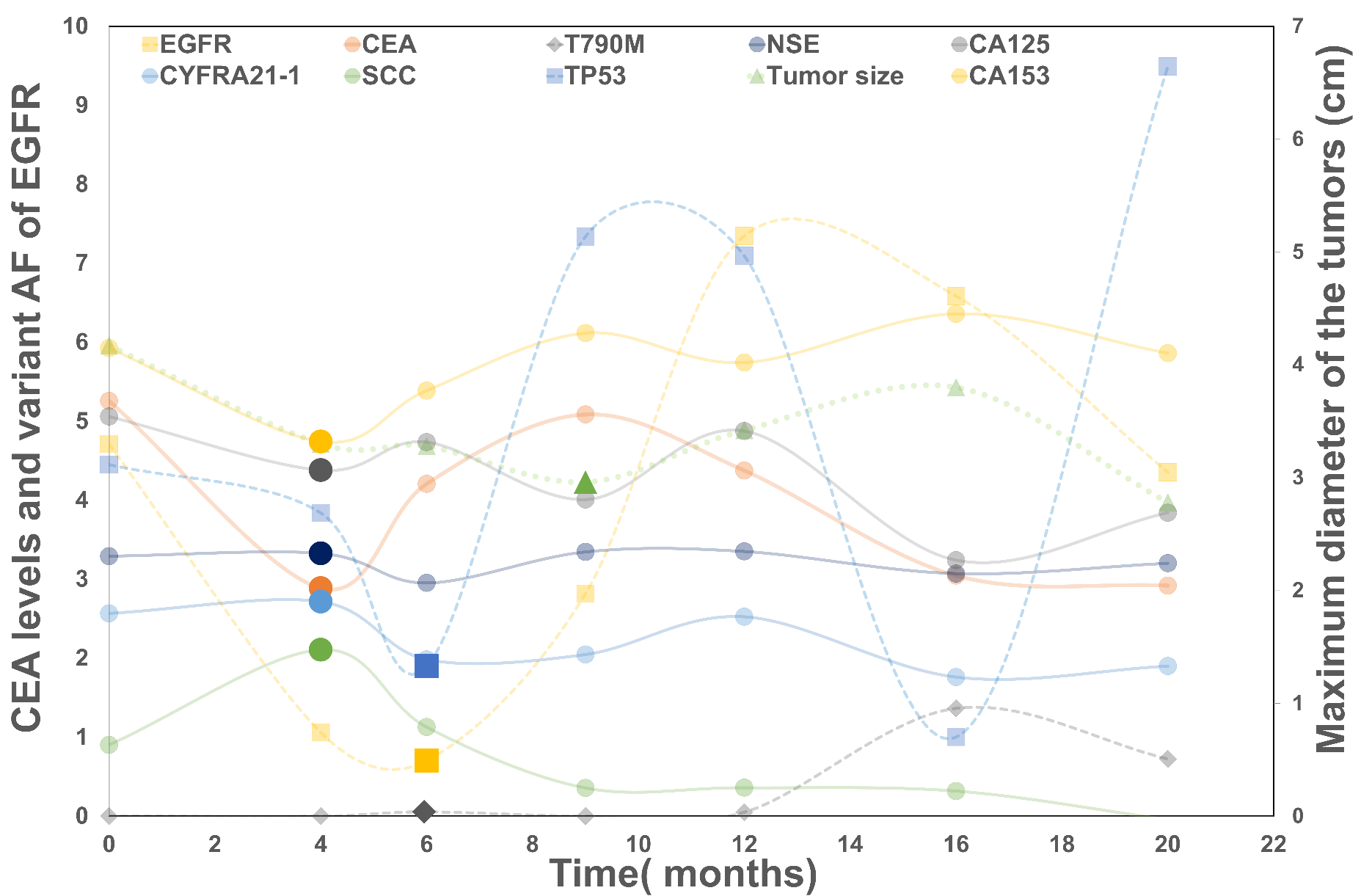


Dynamic changes of tumor size, STMs and ctDNA. Changing trends of STMs levels (The logarithm of the STMs value). Each line represents the average value of patients in the corresponding group. Since the follow-up times for each test were not absolutely synchronous, we used intervals of 2-4 months to compare the dynamic changes between groups. The actual follow-up times for each test are controlled within plus or minus 1 month of the corresponding time point. Darker marks represent the transition time points of each line.

Figure S2. Dynamic variations of the STMs in all patients who resistant to EGFR TKI with negative EGFR T790M or positive EGFR T790M.


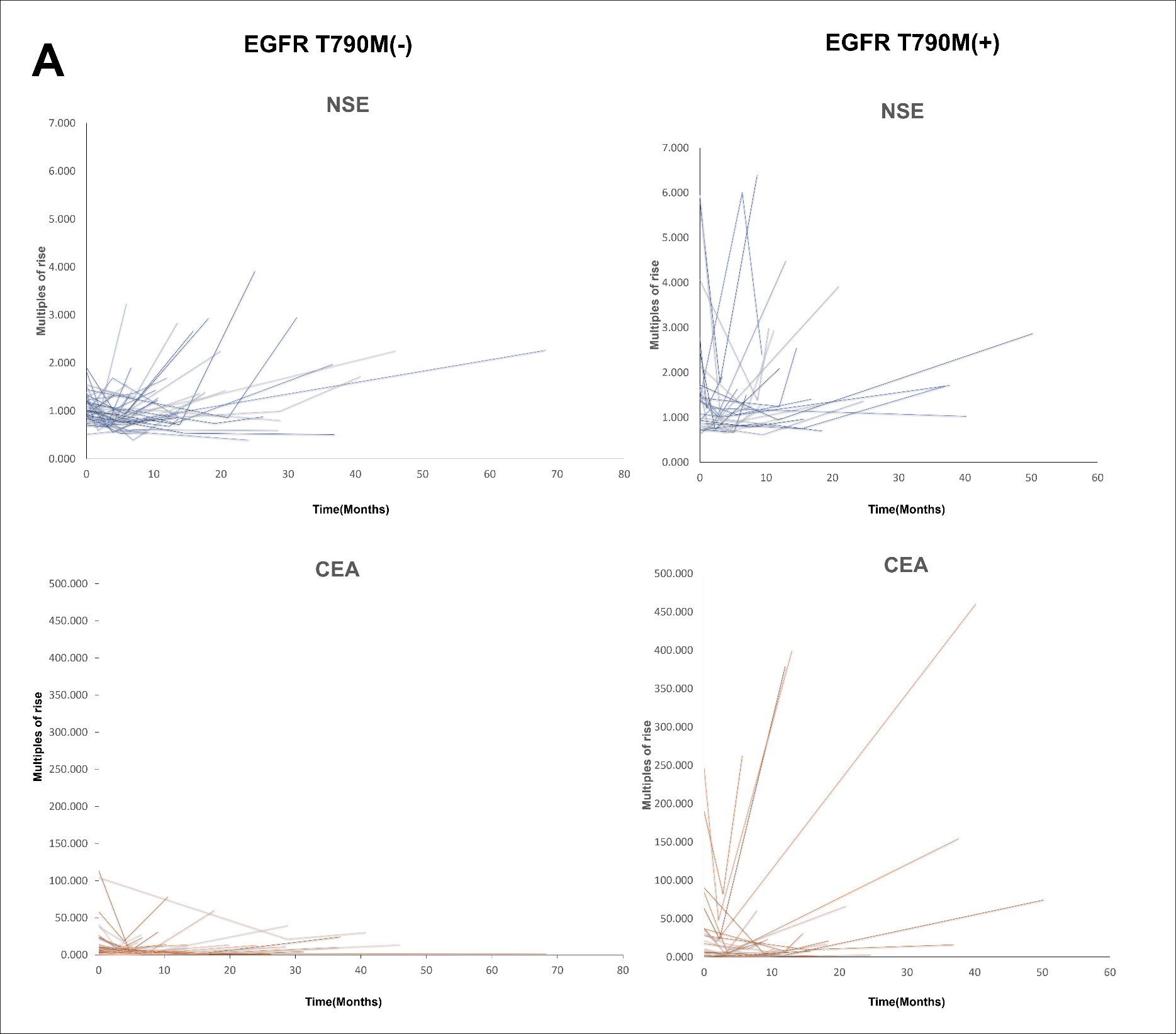

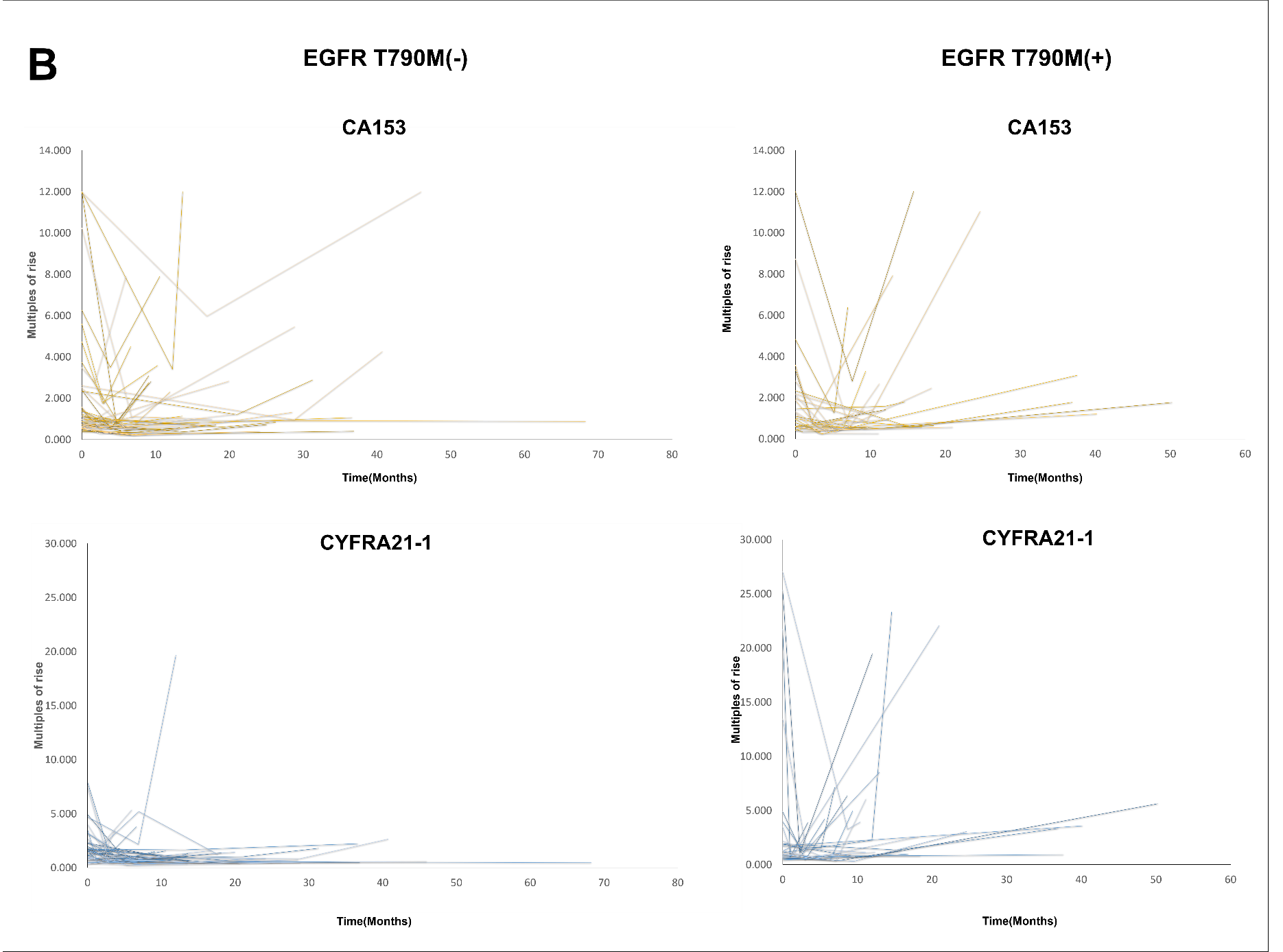

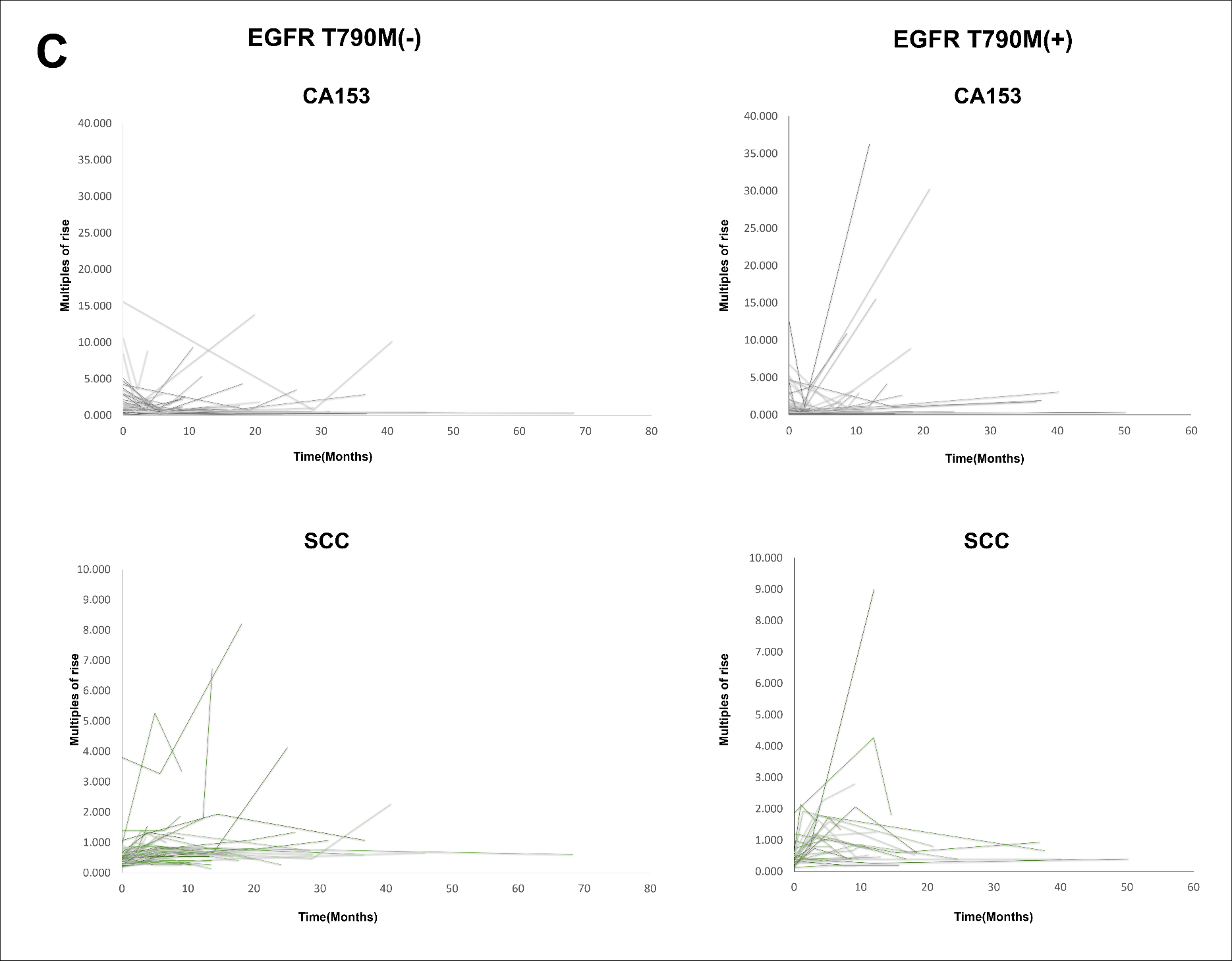


Figure S3. Boxplots of the STMs in the T790M subgroup and the non-T790M subgroup.


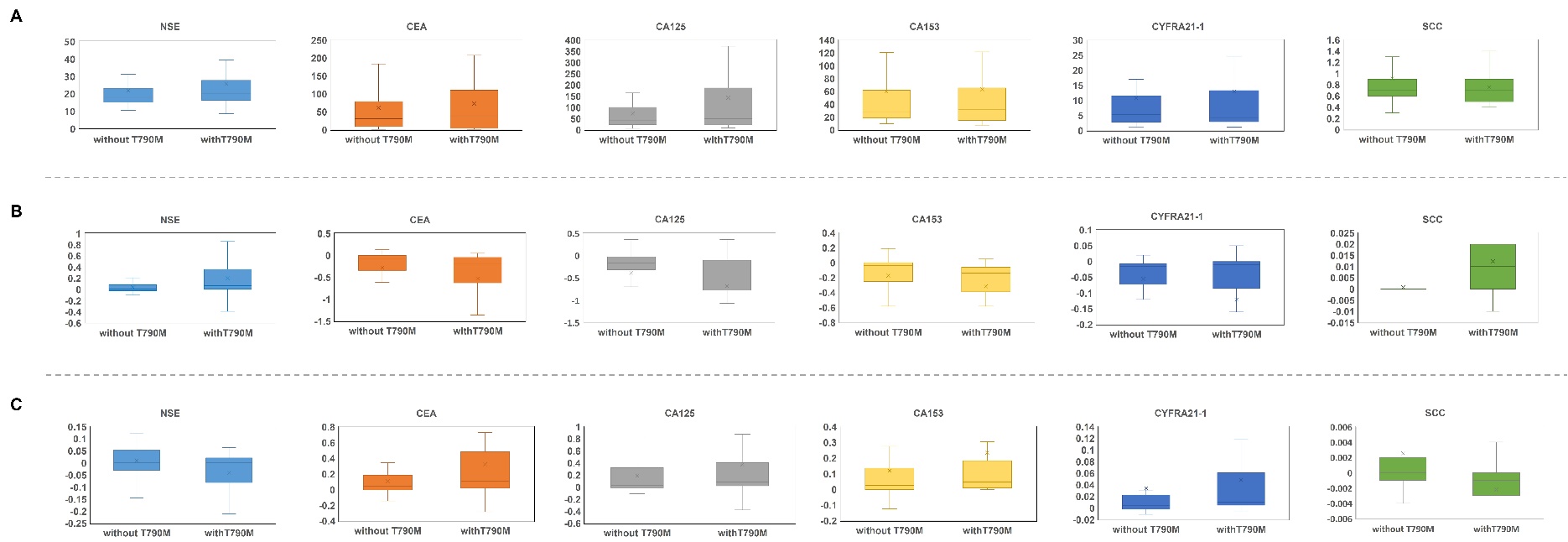


Multiples of the rise of the STMs at baseline(A). Decrease slope of the STMs in the responsive stage(B). Increase slope of the STMs in the resistant stage(C). The box represented the interquartile range (IQR) between the first and the third quartiles, and the midline represented the median. Cross indicated the mean value.

Figure S4. ROC curves of the STMs for predicting secondary TP53 mutation.


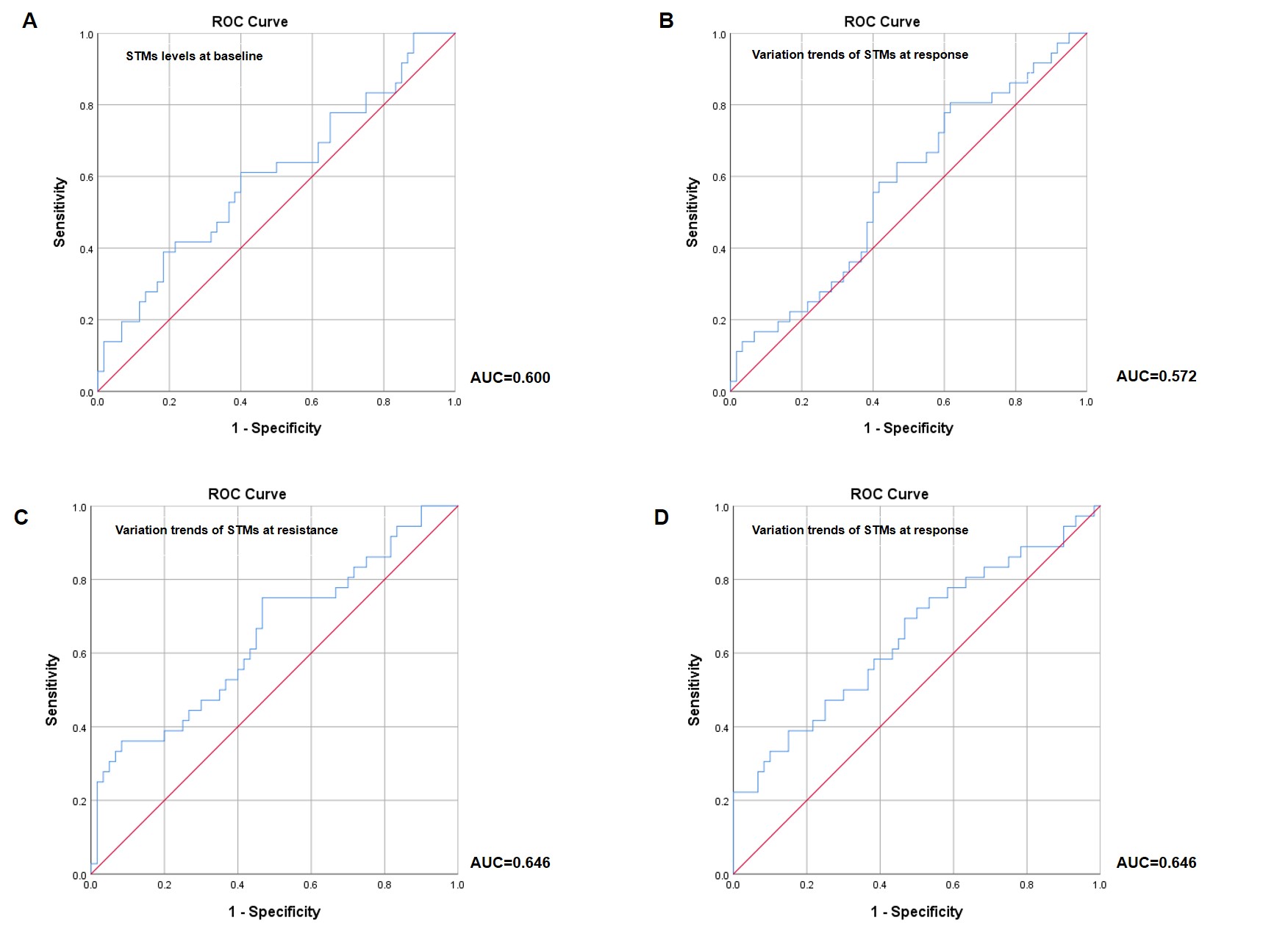


Dynamic STMs poorly predict secondary TP53 mutation. The AUC was 0.600, 0.572, and 0.646 when the STMs levels at baseline(A), the decrease slope of STMs in response(B), and the increase slope of STMs in resistance(C) were analyzed as predictors separately. Besides, the AUC was 0.646 when the STMs levels at baseline and the decrease slope of STMs in response were combined(D).
